# Dermatological ultrasound in assessing skin aging

**DOI:** 10.3389/fmed.2024.1353605

**Published:** 2024-02-12

**Authors:** Ana Luiza Viana Pequeno, Ediléia Bagatin

**Affiliations:** Department of Dermatology, Universidade Federal de São Paulo, São Paulo, Brazil

**Keywords:** ultrasound, skin aging, photoaging, dermatology, technology, high-frequency ultrasound

## Abstract

Ultrasonography (US) has emerged as a pivotal tool in Dermatology since its inaugural use in 1979. Its evolution encompasses technological advancements, higher frequencies, and diverse applications in clinical, surgical, and research aspects. The discussion centers on its crucial role in assessing skin aging through various parameters such as skin thickness, subepidermal low echogenicity band (SLEB) characterization, and echogenicity assessment. This analysis can help guide interventions in a more personalized manner for each patient and assess the effectiveness of cosmetics and procedures. Despite its widespread utility, challenges persist, including discrepancies in research outcomes, operator dependence, inability to detect minute lesions, and measurement variations throughout the day. Combining US with complementary methodologies is advocated for a better understanding of skin aging *in vivo*. The cost-effectiveness and non-invasiveness of the US emphasize its promising future in dermatology, but ongoing research remains imperative to enhance its accuracy and expand its applications.

## 1 Introduction

The advent of ultrasonography (US) in Medicine as a diagnostic imaging modality traces its origins back to the 1940s (1). However, it wasn’t until 1979 that its first application in Dermatology occurred, credited to Alexander and Miller. Their pioneering work utilized a 15 MHz one-dimensional equipment for measuring skin thickness (2). Over the span of more than four decades, dermatological US has undergone remarkable evolution. Advances have led to the development of machines with higher frequencies, aiming to enhance resolution and to visualize superficial structures despite a reduction in wavelength.

Due to its non-invasiveness, painlessness, and cost-effectiveness, dermatological US has been increasingly embraced across various domains within Dermatology (3). Its applications are widespread. In oncology, it aids in early diagnosis, provides critical information about tumor depth, locoregional staging, prognosis, surgical planning and postoperative assessment of treatment outcomes (4). Within clinical Dermatology, US finds utility in the diagnosis of benign tumors, characterizing nail pathology, differentiating autoimmune bullous diseases and monitoring inflammatory conditions such as psoriasis, atopic dermatitis, morphea and hidradenitis suppurativa (5–7). In aesthetic medicine, the US serves as a valuable tool for guiding filler injections, managing complications, and tracking treatment outcomes (3, 8). Moreover, in research, US serves as an objective evaluation parameter across multiple spheres, providing valuable insights into tissue characteristics (2). The utility of US extends notably to the evaluation of photoaging effects on the skin. The potential to characterize ultrasonographic changes in skins of different age groups and within sun-protected and sun-exposed areas represents a pivotal aspect with substantial clinical and research potential (2). This paper aims to centralize its focus on this aspect, highlighting its practical applications, research advancements and future outcomes.

## 2 Principles

US stands as a non-invasive imaging modality that utilizes reflected acoustic waves through tissue to generate images. Its functionality is grounded in the distinct reflection patterns within various tissues, delineating differences in keratin, collagen, and water content. In dermatological applications, the necessity to visualize superficial structures demands higher frequencies (9). This increased frequency directly correlates with improved resolution, enabling finer details to be captured and enhancing the precision of dermatological imaging (10). Transducers are categorized into high (>15 MHz), very high (>20 MHz), and ultrahigh-frequency (30–70 MHz). These devices penetrate between 8 and 30 mm, enabling evaluation of the dermis and subcutaneous layers, or even the epidermis if ultrahigh frequency exceeds 50 MHz (9). The preferred method in Dermatology is B-mode scanning, which converts reflected waves into corresponding ‘brightness’ values displayed on a gray scale (10).

## 3 Skin aging

As individuals age, changes occur in the appearance and features of the skin. Skin aging results from the interplay of intrinsic and extrinsic factors (11). Intrinsic, also referred to as chronological aging, is linked to genetically controlled cellular senescence. Extrinsic factors, recognized as photoaging, stem from environmental elements, notably sun exposure, but also pollution and smoking (11). In this process, the skin becomes thinner and drier, with reduced elasticity and increased irregularity. Wrinkles, dyschromia, and even neoplastic lesions can appear (12). In an effort to mitigate this aging process, there has been an exponential growth in anti-aging cosmetics and aesthetic procedures (13).

The treatment of photoaging begins with the continuous use of sunscreen SPF 30 or higher, applied to all exposed areas, as well as moisturizers that maintain the compromised skin barrier affected by the aging process associated with chronic and uncontrolled sun exposure (14). Several substances have been used in cosmeceuticals to repair signs of aged skin, such as retinoids, antioxidants and hydroxi acids (14). However, the gold standard remains topical 0.05% tretinoin in cream form, with 0.3% adapalene and 0.045% tazarotene also being an option (15–18). Many procedures can be combined with clinical treatment, particularly ablative lasers, such as erbium: yttrium-aluminum-garnet (Er:YAG) and carbon dioxide (CO2); non-ablative lasers, like neodymium: yttrium-aluminum-garnet (Nd:YAG) and Erbium: Glass; and varying depths of chemical peels - superficial, medium, and deep - utilizing substances such as retinoic acid, 35–50% trichloroacetic acid (TCA), and phenol, respectively (19–21).

The multiple therapeutic options available has led to an increasing need for validated methods to evaluate the effectiveness of each treatment (22). These tools should be capable of quantifying and qualifying skin aging, aligning to the requirements of clinical practice as well as research endeavors (22). Regarding clinical scoring systems, mention can be made of the classic and simplified, Glogau scale (1996), and a more recent and detailed one, SCINEXA (2009) (23, 24). However, both are limited by subjectivity and may vary depending on the evaluator.

Objective methods to quantify skin aging are subdivided based on the assessed parameter. Indirect assessments of changes in skin texture and roughness can be made by measuring hydration, including water content in corneal layer and the amount of transepidermal water lost, as aging leads to increased loss of barrier integrity and subsequently higher water loss (22). In this context, notable instruments encompass the TEWLmeter, which assesses transepidermal water loss; the Corneometer, gauging skin electrical capacitance to determine water levels in corneal layer; and *In vivo* confocal Raman spectroscopy, providing quantitative measurements of skin water content (8). For the evaluation of skin firmness, viable tools include the Dermal Torque, Ballistometer, and Cutometer; nevertheless, none have exhibited consistent intra- and interobserver reliability (25). Enhanced assessment of skin pigmentation may be achieved through the utilization of tools such as Video Microscopy and Chromameter; however, these methods still necessitate subjective analysis by the evaluator (8). The gold standard method still remains histopathological analysis, yet it is an invasive approach that may lead to scarring. In this context, the US emerges as a non-invasive sophisticated instrument that provides objective data for more accurate evaluation (26, 27).

## 4 US assessment

Histological data suggest that aged skin exhibits a thinner epidermis compared to younger skin, attributed to the retraction of rete ridges, while the stratum corneum (SC) appears to maintain its normal thickness (2). US studies have shown similar results regarding the SC thickness. However, despite numerous investigations on epidermal, dermal and overall skin thickness, defining the precise changes occurring during the aging process remains challenging (2). Notably, sun-exposed area thickness tends to increase after age 60, while photo-protected areas do not exhibit significant age-related changes (28). Studies utilizing US analyses have demonstrated increased epidermal thickness following the use of retinaldehyde (29), vitamin C (30), and TCA peeling (9). Another study involving ablative CO2 laser treatment for improving photoaging revealed varying outcomes in different areas of the same patient’s face when using identical parameters. Thinner areas where the laser affected the entire thickness exhibited better results compared to thicker areas where the laser only reached the superficial skin layers (31). From this, it can be inferred that US would be an excellent ally in selecting treatment parameters, allowing for customization based on each patient’s skin area and thickness (31).

Other important parameter was identified in 1989 with a characteristic low echogenic band just beneath the epidermis in sun-damaged skin, known as the “subepidermal low echogenicity band” (SLEB) (Supplementary Figure 1) (10). The aging process involves a decreased amount of water in the dermis due, in part, to the loss of the proteoglycans’ hydrophilic properties to retain water. With this alteration, proteoglycans accumulate in the papillary dermis, which reduces echogenicity in that area (30). SLEB thickness rises linearly with age, serving as a valuable marker of photoaging, although it may also be present in non-photoexposed areas. SLEB may undergo changes even throughout the day (2). A study published in 2018 demonstrated the complete disappearance of the SLEB after using 0.05% tretinoin cream for 6 months on the forearms. The investigation utilized the DermaScan (Cortex Technology), an advanced high-frequency US (20 MHz) device for research purposes (32).

The evaluation of echogenicity holds significant importance. Echogenicity typically demonstrates an augmentation associated with aging, in correlation with heightened collagen production and lower amount of intercellular substance (9). Variations in skin echogenicity have been extensively documented in anti-aging treatments, revealing increased echogenicity in both the epidermis and dermis (27). Echogenicity can be objectively assessed by the quantity and mean intensity of pixels (27, 33). Thus, it can be classified into three categories: Low Echogenic Pixels (LEP) ranging from 0 to 30, Medium Echogenic Pixels (MEP) within the 50–150 interval, and High Echogenic Pixels (HEP) between 200 and 255 (9). It becomes feasible to measure the quantity of pixels attributed to LEP, MEP, and HEP. This approach holds potential as an objective assessment method, given the delineation provided in the literature regarding the distinct representations of each of these classes (34, 35). LEP quantifies the level of skin hydration, inflammatory processes, solar elastosis, and collagen degeneration, while MEP and HEP measure the structures of collagen, elastin fibers, and microfibrils (9, 20). Furthermore, the dermis can be subdivided into upper and lower regions, enabling comparison between both and the total. The parameters of the total dermis and upper dermis may prove to be the most reliable and susceptible to reflecting changes in interventions, and thus should be preferred over those of the lower dermis (33).

## 5 Discussion

The use of US in Dermatology has significantly expanded in recent years, encompassing various applications in clinical dermatology, surgical procedures, and research. Within this scope, it serves as an effective tool for assessing skin aging. The primary measures evaluated include skin thickness, characterization of the SLEB, and echogenicity by observing LEP, MEP, and HEP, as well as variations between the upper and lower dermis (2). However, some studies present conflicting results regarding the expected changes in these parameters, highlighting the need for further research to enhance the accuracy of ultrasound methodology for this purpose (9, 33). Until now it is still recommended to combine ultrasonographic evaluation with other clinical or histopathological methods to better assess the outcomes of cosmetics or procedures.

Furthermore, it is important to emphasize the inherent limitations of ultrasound technology. As an operator-dependent imaging method, proper qualifications are essential for optimal use (3). In research, it is advisable to have the same operator perform the assessments throughout the study to avoid biases and to maintain consistent system set parameters, especially the gain (2). Limitations of US technology in the dermatologic field include the inability to detect lesions smaller than <0.1 mm at 15 MHz and <0.03 mm at 70 MHz, or intraepidermal macular lesions such as solar lentigines and café-au-lait macules (3). Concerning the assessment of skin aging, due to variations in measurements throughout the day, efforts to standardize the measurement period in the study are recommended (33).

Considering that it is a non-invasive, cost-effective, and harmless bedside tool, ultrasound holds promising prospects for the future. Transducer technology is continuously improving to enhance the technique. The trend is toward increased popularity among dermatologists and expanded applications for a broader scope of pathologies. However, further research in the field still needs to be intensified.

## Author contributions

AP: Writing – original draft. EB: Conceptualization, Writing – review and editing.
