# Supplementary material for: Dermatological ultrasound in assessing skin aging
Source: Front Med (Lausanne). 2024 Feb 12;11:1353605. doi: 10.3389/fmed.2024.1353605 (PMC10895009; doi:10.3389/fmed.2024.1353605)
Supplement: Supplementary file 1 [file Image_1.pdf]

## *Supplementary Material*

### 1. Supplementary Figures

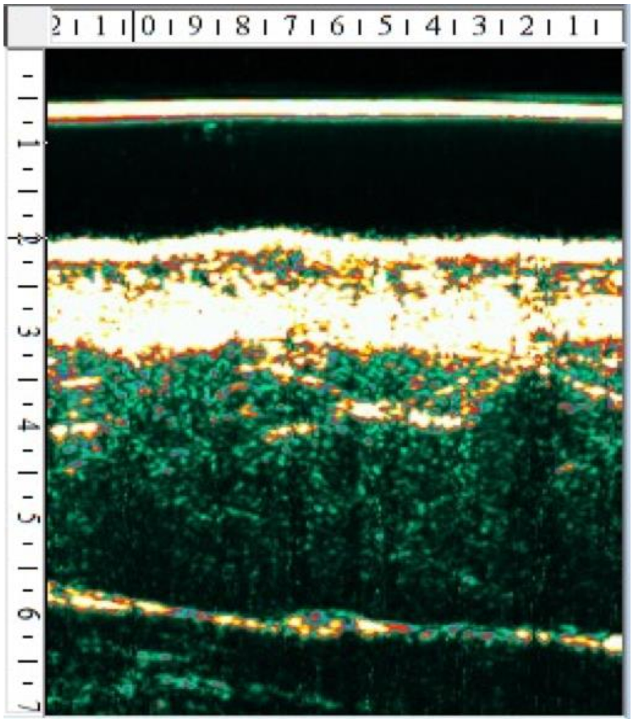

**Supplementary Figure 1.** Images obtained by DermaScan (Cortex Technology) of the forearm skin illustrate the subepidermal low echogenicity band (SLEB).
